# Brain Amyloid Burden and Resting-State Functional Connectivity in Late Middle-Aged Hispanics

**DOI:** 10.3389/fneur.2020.529930

**Published:** 2020-10-06

**Authors:** Mouna Tahmi, Brady Rippon, Priya Palta, Luisa Soto, Fernando Ceballos, Michelle Pardo, Greysi Sherwood, Gabriela Hernandez, Rodolfo Arevalo, Hengda He, Amirreza Sedaghat, Soroush Arabshahi, Jeanne Teresi, Herman Moreno, Adam M. Brickman, Qolamreza R. Razlighi, José A. Luchsinger

**Affiliations:** ^1^Department of Medicine, College of Physicians and Surgeons, Columbia University Irving Medical Center, New York, NY, United States; ^2^Department of Epidemiology, Joseph P. Mailman School of Public Health, Columbia University Irving Medical Center, New York, NY, United States; ^3^Department of Biomedical Engineering, Columbia University, New York, NY, United States; ^4^Research Division, Hebrew Home in Riverdale, Bronx, NY, United States; ^5^Departments of Neurology and Physiology/Pharmacology, The Robert F. Furchgott Center for Neural and Behavioral Science, SUNY Downstate Medical Center, New York, NY, United States; ^6^Kings County Hospital Neurology, New York, NY United States; ^7^Department of Neurology, College of Physicians and Surgeons, Columbia University Irving Medical Center, New York, NY, United States; ^8^Taub Institute for Research on Alzheimer's Disease and the Aging Brain, Columbia University Irving Medical Center, New York, NY, United States; ^9^Gertrude H. Sergievsky Center, Columbia University Irving Medical Center, New York, NY, United States; ^10^Department of Radiology, Weill Cornell Medicine, New York, NY, United States

**Keywords:** Alzheimer's disease, amyloid beta, RsfMRI, fMRI, functional connectivity, middle-age, hispanic

## Abstract

Non-linear relations of brain amyloid beta (Aβ) with task- based functional connectivity (tbFC) measured with functional magnetic resonance imaging (fMRI) have been reported in late middle age. Our objective was to examine the association between brain Aβ and resting-state functional connectivity (rsFC) in late middle-aged adults. Global brain Aβ burden was ascertained with ^18^F-Florbetaben Positron Emission Tomography (PET); rsFC was ascertained on 3T Magnetic Resonance Imaging (MRI) among 333 late middle-aged Hispanics adults without dementia in four major brain functional connectivity networks: default mode network (DMN), fronto-parietal control network (FPC), salience network (SAL) and dorsal attention network (DAN). We examined the relationship of global brain Aβ with rsFC using multivariable linear regression adjusted for age, sex, education, and APOE-ε4 genotype. We quantified the non-linear associations both with quadratic terms and by categorizing Aβ into three groups: low Aβ, intermediate Aβ, and positive Aβ. We found no significant linear or non-linear associations between Aβ, measured either continuously or categorically, with rsFC in the examined networks. Our null findings may be explained by the younger age of our participants in whom amyloid burden is relatively low. It is also possible that the recently reported non-linear relationship is exclusive to task fMRI and not rsfMRI.

## Introduction

The deposition of amyloid beta (Aβ) in the brain starts decades before it impacts cognition (1). The effects of Aβ on brain function using resting-state functional connectivity (rsFC) magnetic reasoning imaging (fMRI) have been largely studied in cognitively normal older adults and have yielded conflicting findings with both positive and negative linear relationships reported for Aβ levels and functional connectivity (2–6). Similar results of hyperactivation and hypoactivation in association with brain Aβ are also observed with task activation (7–10). To address these mixed results, a recent study of 62 middle-aged and older adults (mean age: 67.73 years) reported a non-linear quadratic association between levels of Aβ and task-based functional connectivity (tbFC), such that intermediate levels of Aβ are associated with higher activation in regions of the default mode network (DMN), but high levels of Aβ are associated with lower activation (11). Another study from the same group (*n* = 68, mean age: 68.59) replicated these findings using a different functional task paradigm, suggesting that this non-linear relationship between Aβ and FC is task-independent (12). If replicated in rsFC, such a finding would provide evidence for the hypothesis that the brain dose-dependent response to levels of Aβ may also be apparent at rest when no task is performed (12).

Our primary goal in this study was to determine if the non-linear association of Aβ with functional connectivity, recently observed in tbFC, is also present at rest using fMRI in late middle-age, a critical period for the deposition of Aβ (13). Thus, we examined the association of global brain Aβ burden with rsFC among 333 late middle-aged Hispanic adults without dementia in four major cognitive networks: default mode network (DMN), fronto-parietal control network (FPC), dorsal attention network (DAN), and salience network (SAL).

## Materials and Methods

### Participants

**Figure 1:**
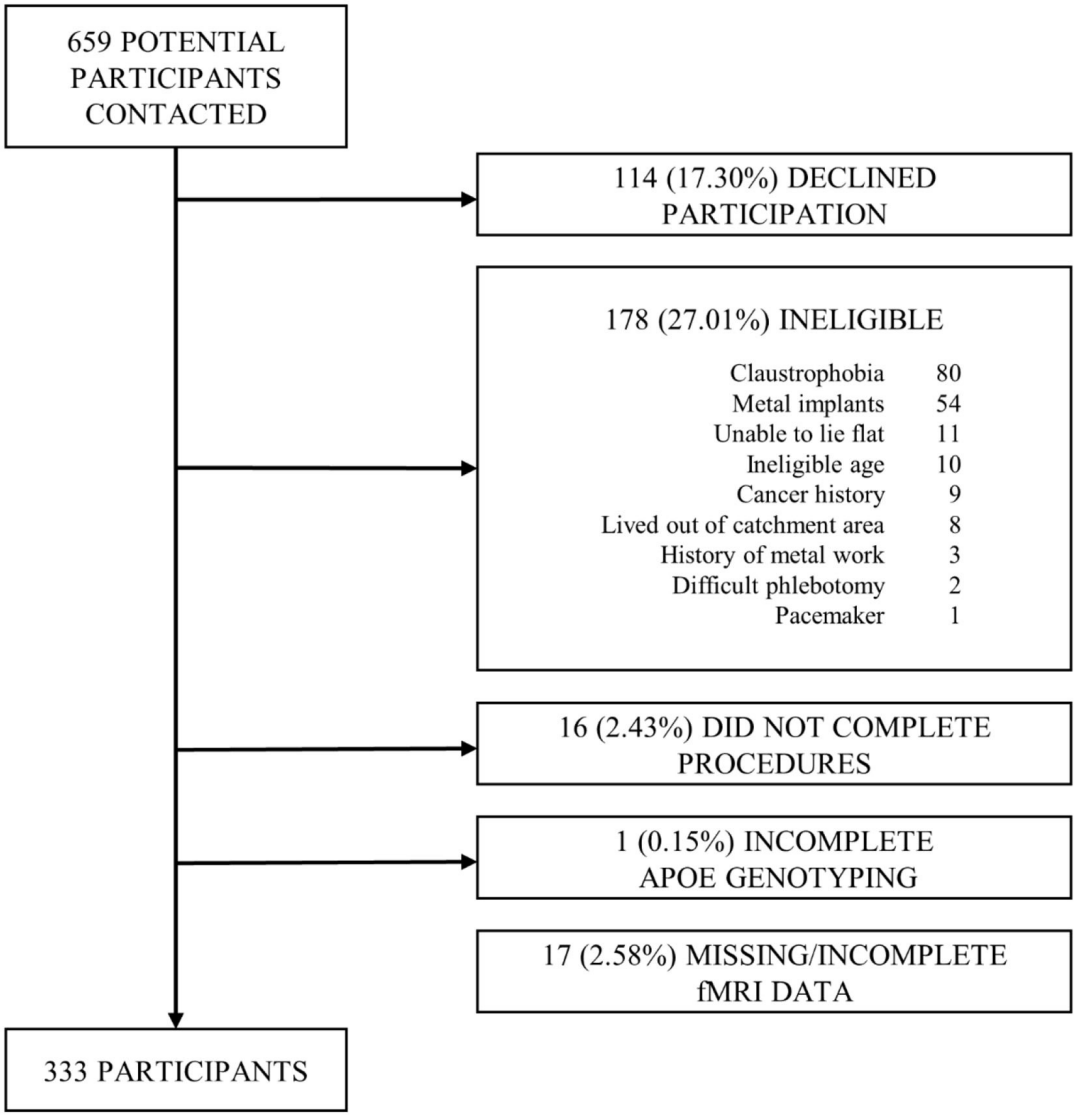
Flow chart of participant recruitment.

This was a cross-sectional analysis of a community-based cohort of 333 late middle-aged Hispanic adults without dementia and with functional connectivity data out of 350 recruited between 03/01/2016 and 07/31/2019 in New York City as part of a study of Alzheimer's disease biomarkers (14). We targeted Hispanics because they are the most common ethnic group in the community surrounding CUIMC (15) and because there is a paucity of AD biomarkers studies in Non-Whites (13). Inclusion criteria included ages between 55 and 69 years, men and women, able to undergo phlebotomy, clinical and neuropsychological assessments, 3T brain magnetic resonance imaging (MRI), and Positron Emission Tomography (PET) with the Aβ radioligand ^18^F-Florbetaben. Exclusion criteria included dementia diagnosis, cancer other than non-melanoma skin cancer, and MRI contraindications. We screened 659 potential participants; 114 (17.30%) declined to participate, 178 (27.01%) were ineligible, 16 (2.43%) did not complete study procedures; 17 participants (2.58%) were excluded due to incomplete fMRI data and one additional participant (0.15%) was excluded from analyses due to incomplete data on APOE genotype, the most important predictor of *in-vivo* brain amyloid burden (16) (Figure 1). The interval between amyloid PET and MRI was 15.79 ± 33.41 days. The Institutional Review Board and the Joint Radiation Safety Commission at CUIMC approved this study. Participants provided written informed consent. Funding sources had no role in study design, data collection, data analyses or interpretation.

### Exposure: Global Brain Aβ

Our main exposure of interest was global brain Aβ ascertained with ^18^F-Florbetaben positron emission tomography (PET). The dose of ^18^F-Florbetaben was 300 MBq (8.1 mCi), maximum 30 mcg mass dose, administered as a single slow intravenous bolus. Images were acquired over 20 min starting 90 min after injection. Dynamic PET frames (4 scans) were aligned to the first frame using rigid-body registration and a static PET image was obtained by averaging the four registered frames. Additional information on the Aβ PET scan processing protocol has been previously published (17). The standardized uptake value (SUV), defined as the decay-corrected brain radioactivity concentration normalized for injected dose and body weight, was calculated in all regions and each voxel, which subsequently normalized to the SUV in cerebellar gray matter to derive the regional and voxel-wise SUVR. We used a standard approach to calculating global Aβ burden using Alzheimer's Disease Neuroimaging Initiative (ADNI) defined regions of interests (ROIs) to generate four lobar SUVRs (frontal, temporal, cingulate and parietal cortex) (18). We then averaged them to compute an overall mean value of global cortical Aβ. Aβ measures were examined both continuously and categorically. We examined the non-linear association of Aβ measured continuously by incorporating quadratic terms in the model. The threshold for dichotomization into Aβ positive and negative groups was 1.342 and was derived using the K-means clustering method. To classify two groups, the K-means clustering algorithm starts by identifying two Aβ SUVR values from our data at random and subsequently assigns all other observations to the group with the closest mean Aβ SUVR. Once this process has been completed for all observations, it continues to reiterate, checking if observations should be reclassified to the other cluster, until all group assignments are stable. The final classifications should minimize each within-cluster variance, equivalent to the sum of the squared Euclidean distance between each Aβ SUVR value and its within-cluster mean (19). Values under this resulting threshold (SUVR = 1.342) were further dichotomized as intermediate and low using a median split (SUVR = 1.126).

### Outcome: Resting-State Functional Connectivity

Resting-state scans were collected on a 3T GE scanner during 10 min of resting state protocol where participants were instructed to lie still, keep their eyes open, and to not think about anything in particular. T1-weighted images of the whole brain were acquired for each participant with a magnetization prepared rapid gradient-echo sequence (Time of Inversion (TI) = 450 ms; Flip angle (FA) =12 degree; Field of view (FOV) = 25.6 × 25.6 cm; Matrix size = 256 × 256). Ten minutes of rsfMRI scans were collected with GRE-EPI sequence using the following parameters: TR/TE = 2,000/23 ms; FOV =19.2 × 19.2 cm; FA = 77 degree; matrix size = 96 × 96; slice thickness/gap = 3/0 mm; number of slices = 40.

### fMRI Data Processing

Images were preprocessed using an in-house developed native space method (20). Briefly, “slice timing correction was done using the FSL slice timing tool (21). We then used *mcflirt* (a motion correction tool available in the FSL package) to register all volumes to a reference image (21, 22). The reference image was generated by registering (6 df, 256 bins mutual information, and *Sinc* interpolation) all volumes to the middle volume and averaging them. We then used the method described in Power et al. (23) to calculate frame-wise displacement (FWD) from the six motion parameters and root mean square difference (RMSD) of the realigned fMRI signal in the consecutive volumes for each subject. To be conservative, we lowered the threshold of our RMSD to 0.3% (it was originally suggested to be 0.5%). RMSD was computed on the motion-corrected volumes before temporal filtering. The contaminated volumes were detected by the criteria FWD > 0.5 mm or RMSD > 0.3%. Identified contaminated volumes were replaced with new volumes generated by linear interpolation of adjacent volumes. Volume replacement was done before band-pass filtering (24). The motion-corrected signals were passed through a band-pass filter with the cutoff frequencies of 0.01 and 0.09 Hz. We used *flsmaths– bptf* to do the filtering in this study (22). Finally, we residualized the motion-corrected, scrubbed, and temporally filtered volumes by regressing out the FWD, RMSD, left and right hemisphere white matter, and lateral ventricular signals (25).”

T1-weighted image segmentation was performed with FreeSurfer (http://surfer.nmr.mgh.harvard.edu/) (26–28) and inspected visually for any inaccuracies. Using the state of the art non-linear registration method, advanced normalization tools (ANTs), we transferred the 264 putative functional coordinates derived from a brainwide graph described by Power et al. (29) from MNI space to subjects' native space. A conjunction mask was obtained as an intersection of a 10 mm radius spherical mask, centered at each coordinate and the native space gray-matter mask. This conjunction mask was used to compute the time-series for each coordinate by averaging the time-series of all voxels that fall within the conjunction mask. To prevent inflation of within-network functional connectivity (correlation), we also averaged all coordinates that fell within each node of the networks. For instance, all the DMN coordinates that were closely located in the left/right angular gyrus and inferior parietal regions were averaged to obtain a single time-series for left/right angular gyrus node of the DMN. This resulted in 53 separate nodes representing all the networks reported in Power et al. (29). The averaged functional time-series of each of the 53 regions were used to compute a correlation matrix for each participant preprocessed rsfMRI scan. Then, the correlations were Fisher z-transformed to generate normalized correlation matrices for each subject. In order to remove correlations between an area and itself, the diagonal of each correlation matrix was set to zero. Regions of interest (ROIs) were labeled based on their network assignment according to Power et al. (29) and within network functional connectivity was computed by averaging the inter-nodal correlation within each network. Four networks were preselected for statistical analysis based on their role on cognitive processes: default mode network (DMN; 9 nodes), salience network (SAL; 9 nodes), fronto-parietal control network (FPC; 9 nodes), and dorsal attention network (DAN; 6 nodes). For details on the location of each node within each network, see Supplemental Table 1.

### Neuropsychological and Behavioral Assessment

All participants were administered a neuropsychological assessment that included tests of memory, language, visuospatial abilities, and processing speed. A Spanish version of the neuropsychological test battery was used for Spanish-speaking participants (30). The following tests were included: the Buschke Selective reminding Tests (SRT) and its components, including total recall, delayed recall, and delayed recognition (31); the Boston Naming-15 item Test (32); letter fluency; category fluency; the similarities subtest from the Wechsler Adult Intelligence Scale-Revised (33); the repetition and comprehension subtests of the Boston Diagnostic Aphasia Evaluation (34); the recognition and matching components of the Benton Visual Retention Test (35); the Rosen Drawing Test (36); the identities and oddities subtest from the Mattis Dementia Rating Scale (37); Color Trails 1 and 2 (38). To aggregate the neuropsychological test data, we performed a Principal Component Analysis (PCA). PCA is a statistical method most commonly used for data reduction on a set of collinear variables, where each principle component is a linear combination of the centered loading variables (in this case, our neuropsychological test scores) such that the variance of these variables is maximized (39). Each principle component is independent and captures a proportion of the variance in the neuropsychological tests, meaning that 15 principle components would completely capture the variance in our 15 tests. We chose to only retain the first principle component for our analysis as it captured much more variance in our tests than any other component. Additionally, the difference in the variance captured by all other principle components was not appreciable by visual inspection of the scree plot of our components (Supplemental Figure 1).

### Covariates

Characteristics considered as potential covariates included age, sex, education, and APOE-ε4 genotype. We controlled for age, sex and education due to their known effects on functional connectivity (40, 41). We controlled for APOE-ε4 genotyping because it is a strong determinant of Aβ burden (42). Participants were classified as APOE-ε4 carriers if they were homozygous or heterozygous for APOE-ε4. APOE-ε4 genotyping was conducted by LGC genomics (Beverly, MA) using single nucleotide polymorphisms rs429358 and rs7412.

### Statistical Analysis

RsFC values in the four networks examined were right-skewed and required logarithmic transformation to approximate a normal distribution. Bivariate comparisons across Aβ categories were made using analysis of variance (ANOVA) for continuous variables. Differences in categorical variables were evaluated using chi-squared tests. The relationship between global brain Aβ SUVR and rsFC was evaluated with a multivariable linear regression model including continuous Aβ SUVR and squared terms for Aβ SUVR, adjusted for age, sex, education, and APOE-ε4 carrier status. The relationship of Aβ categories (low, intermediate, and positive) with rsFC was examined with linear regression models adjusted for age, sex, education, and APOE-ε4 carrier status. The statistical significance was defined as *p* < 0.05. All statistical analyses were performed using the Statistical Package for the Social Sciences (SPSS) 26 (SPSS, Chicago, IL) and R version 3.6.0.

### Sensitivity Analyses

As a sensitivity analysis, we examined the association of Aβ with rsFC among participants who were cognitively normal only. Cognitive impairment was defined according to cutoffs of 1.0 or 1.5 standard deviations below the first principal component of the neuropsychological battery of our sample (43). The rationale for conducting sensitivity analyses restricted to cognitively normal participants was to replicate prior studies, which exclusively examined these associations among cognitively normal participants (11, 12). We validated our assumption that the first principle component of the neuropsychological battery is an appropriate approximation of global cognition by verifying that better performance on all neuropsychological evaluations was significantly positively correlated with increases in this principle component.

## Results

**Figure 2:**
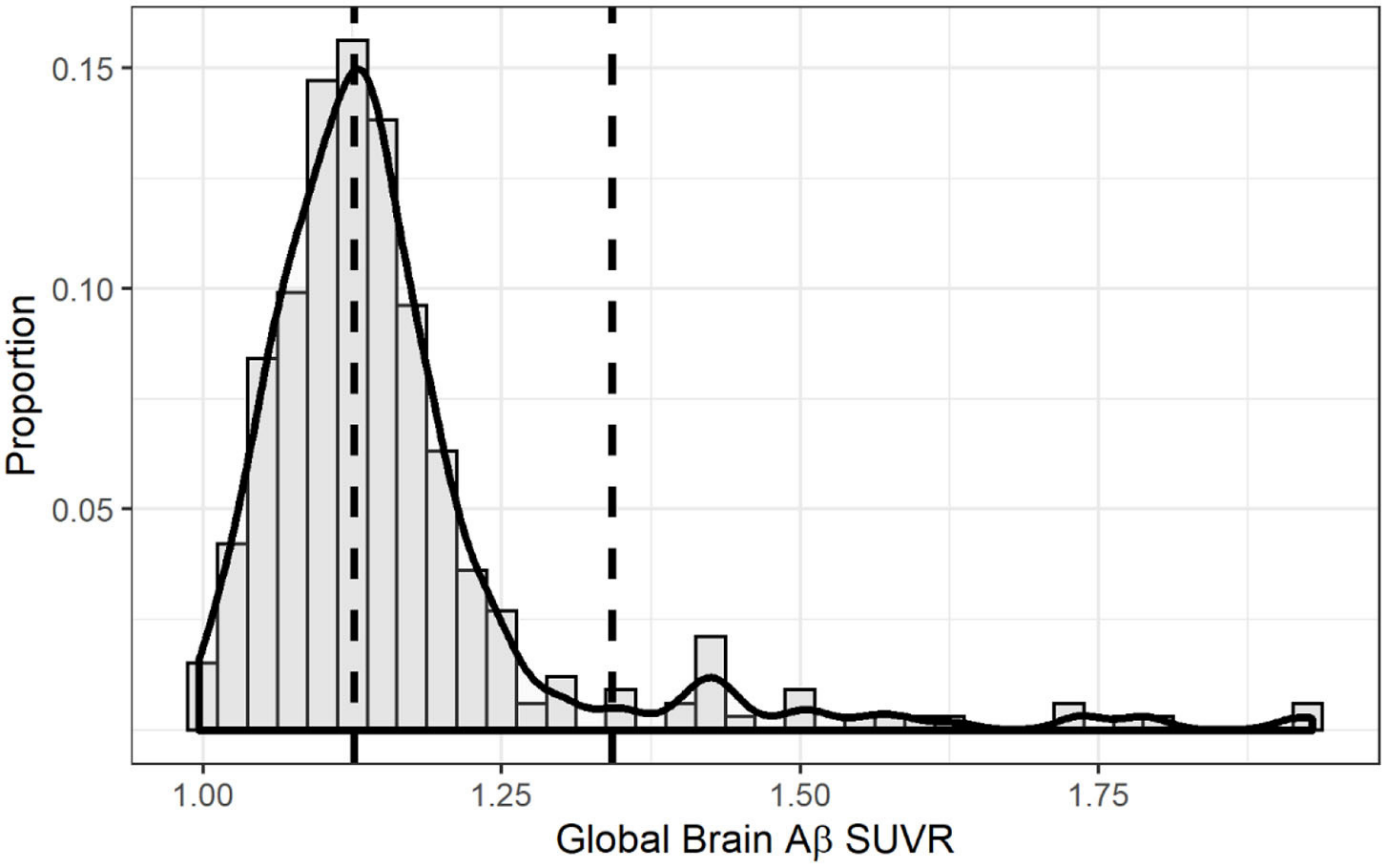
Distribution of global brain amyloid SUVR. The solid line overlaid onto the histogram represents a non-parametric kernel density estimation. The right vertical dashed line marks the threshold for amyloid positivity using the k-means clustering method (1.342) while the left vertical line marks the threshold for intermediate amyloid (1.126).

Table 1 shows the characteristics of the total sample and across Aβ categories (low, intermediate, positive). The sample consisted of 237 women and 96 men, who had a mean age of 64.14 ± 3.37 years and 10.51 ± 3.95 years of education. There were no significant differences across Aβ groups with respect to age and education. There was a higher proportion of women among the intermediate and positive Aβ categories. As expected, compared to participants without the APOE-ε4 allele, APOE-ε4 carriers had higher odds of being in the intermediate or positive category as compared to the low Aβ category (Table 1, Supplemental Figure 2). Figure 2 shows the distribution of global Aβ SUVR in the sample. As expected, Aβ SUVR had a bimodal distribution and the first peak resembled a normal distribution.

**Table 1:** Characteristics of the entire sample and by amyloid β (Aβ) category.

	**Total sample (*n* = 333)**	**Low Aβ (*n* = 154)**	**Intermediate Aβ (*n* = 153)**	**Positive Aβ (*n* = 26)**	***P*-value^a^**
Age, mean (SD), y	64.14 (3.37)	63.76 (3.54)	64.40 (3.19)	64.83 (3.27)	0.14
Sex, Women, *N* (%)	237 (71.17)	86 (55.84)	132 (28.27)	19 (73.08)	<0.0001
Education, mean (SD), y	10.51 (3.95)	10.48 (3.77)	10.34 (4.15)	11.62 (3.80)	0.31
Ethnicity, *N* (%)					0.76
Dominican	286 (85.88)	132 (85.71)	134 (87.58)	20 (76.92)	
Other Caribbean Hispanic	20 (6.0)	9 (5.84)	8 (5.23)	3 (11.54)	
South American	17 (5.10)	9 (5.84)	6 (3.92)	2 (7.69)	
Central American	4 (1.20)	1 (0.65)	3 (1.96)	0 (0)	
Unspecified Hispanic	3 (0.90)	2 (1.30)	1 (0.65)	6 (23.08)	
Spanish speakers, *N* (%)	312 (93.69)	144 (93.51)	144 (94.12)	24 (92.31)	0.93
APOE-ε4 carriers, *N* (%)^a^	117 (35.13)	40 (25.97)	56 (36.60)	21(80.77)	<0.0001

*SD, standard deviation; Aβ, Amyloid beta*.

a *Bivariate comparisons across Aβ categories were made using analysis of variance (ANOVA) for continuous variables. Differences in categorical variables were evaluated using chi-squared tests*.

**Figure 3:**
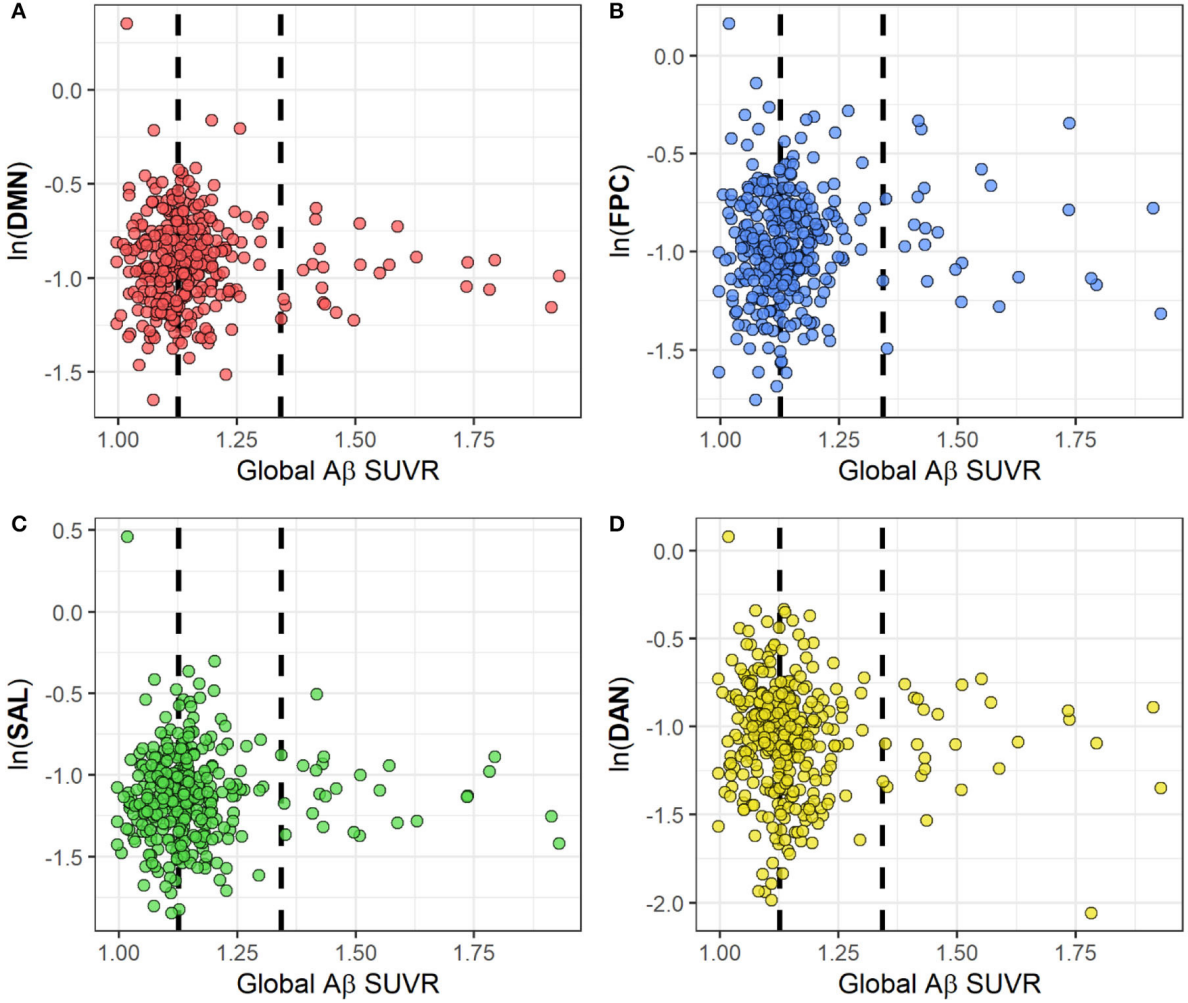
Scatterplots for the association between rsFC and global Aβ SUVRs in four cognitive networks: DMN **(A)**, FPC **(B)**, SAL **(C)** and DAN **(D)**. RsFC for each network has been log-transformed. The two dashed lines represent Aβ thresholds between low and intermediate (SUVR = 1.126) and between intermediate and positive (SUVR = 1.342) categories.?Abbreviations: Ln, natural logarithm; Aβ, amyloid beta; SUVR, Standardized Uptake Value Ratio; rsFC, resting state functional connectivity; DMN, default mode network; FPC, fronto-parietal control; DAN, dorsal attention network; SAL, salience.

Figure 3 represents scatter plots for the association between rsFC in the four networks and global Aβ SUVR. We observed no linear or quadratic association between Aβ and rsFC in the four networks, including the DMN. Results for adjusted and unadjusted associations with continuous Aβ (SUVR) and rsFC are reported in Table 2. In Table 3, we report associations between dichotomized Aβ categories and rsFC. Results were similar in sensitivity analyses restricted to participants who were cognitively normal only based either on 1.5 (*n* = 318) or 1.0 SD (*n* = 291) below the first PCA of the sample neuropsychological performance.

**Table 2:** Coefficients and *p*-values from linear regression for linear and quadratic term of Aβ (SUVR) on rsFC.

	**Aβ** **(SUVR) linear term**		**Aβ** **(SUVR) quadratic term**	
	**β (CI)**	***P***	**β (CI)**	***P***
**Model 1**				
LnDMN	0.93 (−1.00, 2.85)	0.35	−0.37 (−1.07, 0.34)	0.31
LnFPC	−1.46 (−0.77, 3.69)	0.20	−0.49 (−1.31, 0.33)	0.24
LnDAN	−0.95 (−3.50, 1.60)	0.46	0.28 (−0.66, 1.22)	0.55
LnSAL	1.35 (−0.87, 3.57)	0.23	−0.48 (−1.30, 0.33)	0.25
**Model 2**				
LnDMN	0.25 (−1.72, 2.22)	0.80	−0.13 (−0.85, 0.59)	0.72
LnFPC	0.88 (−1.41, 3.17)	0.45	−0.30 (−1.13, 0.54)	0.48
LnDAN	−0.37 (−3.02, 2.27)	0.78	0.10 (−0.87, 1.06)	0.84
LnSAL	1.15 (−1.11, 3.41)	0.32	−0.39 (−1.21, 0.35)	0.36

*Ln, natural logarithm; Aβ, amyloid beta; SUVR, Standardized Uptake Value Ratio; rsFC, resting state functional connectivity; DMN, default mode network; FPC, fronto- parietal control; DAN, dorsal attention network; SAL, salience*.

*Model 1 is unadjusted. Model 2 is adjusted for age, sex, education and APOE-ε4*.

**Table 3:** Association between Aβ categories (low, intermediate and positive) and rsFC using linear regressions.

	**Low to intermediate Aβ**		**Low to positive Aβ**		**Intermediate to positive Aβ**		***P*-trend**
	**β (CI)**	**P**	**β (CI)**	**P**	**β (CI)**	**P**	
**Model 1**							
LnDMN	0.04 (−0.01, 0.10)	0.13	−0.05 (−0.15, 0.05)	0.36	−0.09 (−0.19, 0.01)	0.08	0.12
LnFPC	0.03 (−0.03, 0.10)	0.27	0.07 (−0.04, 0.19)	0.21	0.04 (−0.08, 0.16)	0.51	0.33
LnDAN	−0.05 (−0.12, 0.02)	0.19	−0.07 (−0.20, 0.07)	0.32	−0.02 (−0.15, 0.11)	0.78	0.34
LnSAL	0.04 (−0.02, 0.10)	0.20	0.05 (−0.07, 0.16)	0.42	0.007 (−0.11, 0.12)	0.91	0.40
**Model 2**							
LnDMN	0.02 (−0.04, 0.07)	0.57	−0.05 (−0.16, 0.05)	0.31	−0.07 (−0.17, 0.03)	0.18	0.39
LnFPC	0.01 (−0.05, 0.08)	0.69	0.05 (−0.07, 0.18)	0.40	0.04 (−0.08, 0.05)	0.69	0.69
LnDAN	−0.02 (−0.10, 0.05)	0.54	−0.03 (−0.18, 0.11)	0.64	−0.01 (−0.15, 0.13)	0.89	0.79
LnSAL	0.04 (−0.03, 0.10)	0.29	0.09 (−0.03, 0.21)	0.15	0.05 (−0.06, 0.17)	0.37	0.29

*Ln, natural logarithm; Aβ, amyloid beta; SUVR, Standardized Uptake Value Ratio; rsFC, resting state functional connectivity; DMN, default mode network; FPC, fronto- parietal control; DAN, dorsal attention network; SAL, salience*.

*Model 1 is unadjusted. Model 2 is adjusted for age, sex, education and APOE-ε4*.

## Discussion

Our goal in the current study was to test if the recent evidence of a dose-dependent relationship between Aβ and tbFC in regions of the DMN (11, 12) are also present at rest. We found that there is no association between Aβ burden and rsFC.

In a study of 62 middle-aged and older adults (mean age=67.73 years), Foster et al. (11) reported that the association between Aβ SUVR and tbFC is quadratic with both hyperactivation and hypoactivation phases in regions of the DMN. These findings were further replicated by the same group using a different functional task paradigm (*n* = 68, mean age: 68.59), suggesting that this non-linear relationship is task independent (12). However, the question on whether this observed association between Aβ and functional connectivity is actually present at rest remains unanswered. The results from our large sample of 333 found no linear or non-linear association between global brain Aβ and functional connectivity in major cognitive networks, including DMN, measured at rest.

There are several possible explanations of our findings. First, it is possible that the reported non-linear association by Foster et al. with task fMRI (11, 12), although independent of the type of task performed, may be dependent on the action of performing a task in general and therefore not an intrinsic effect apparent at rest. The fact that this was observed using different task paradigms does not entirely confirm its task independency. Alternatively, it is possible that the relatively younger age group of our sample is at its initial stages of Aβ accumulation which has not yet affected rsFC. Most of the reported studies on Aβ and rsFC were done in older adults and still yielded different results between a positive and negative associations (2–6). A null association with static rsFC, as was observed in our study, was also reported in a community-based cohort of 133 adults with a mean age of 72 years (44). A follow-up of our cohort will allow for more time for Aβ accumulation, at which point an effect on rsFC may be detected. Finally both Aβ and tau accumulation may be required for the non-linear effects when it comes to the resting brain which has been recently reported in cognitively normal older adults (45). We cannot address the effects of tau in our data. However, we are currently collecting tau PET data in this sample, which will allow us to investigate the interactive roles of tau and Aβ on functional connectivity in future analyses.

Strengths of our study include the relatively large sample of late middle-aged Hispanic adults within a relatively narrow age range, which addresses a gap in studies in late middle age and in ethnic minorities. The main limitation of our study is its cross-sectional design, which limits our ability to infer causality from our findings. It is also possible that our findings are not generalizable to other ethnic groups. However, we could also argue that our findings may be generalized to other ethnics groups since APOE-ε4 in our sample was associated with higher amyloid burden as reported in most Non-Hispanic White samples (46).

In conclusion, we found that no association between brain Aβ burden and rsFC in our cohort of late middle-aged Hispanics without dementia. Continued follow-up of the same cohort is needed to examine future effects of Aβ accumulation on brain functional networks.

## Data Availability Statement

The datasets generated for this study are available on request to the corresponding author.

## Ethics Statement

The studies involving human participants were reviewed and approved by the Institutional Review Board at Columbia University Irving Medical Center. The patients/participants provided their written informed consent to participate in this study.

## Author Contributions

MT conducted statistical analyses, and drafted the initial manuscript. BR performed data curation, advised on data interpretation, and manuscript review. PP advised on data interpretation and manuscript review. LS, FC, MP, GS, GH, and RA assisted with data collection. HH conducted the PET data processing. AS conducted the fMRI data processing. SA helped with fMRI data processing. JT, HM, and AB advised on data interpretation and manuscript review. QR advised on fMRI/PET data analyses and manuscript review. JL advised on data interpretation and manuscript review and supervised the project. All authors contributed to the article and approved the submitted version.

## Conflict of Interest

JL receives a stipend from Wolters Kluwer, N.V. as Editor in Chief of The Journal Alzheimer's Disease and Associated Disorders, and has served as a paid consultant to vTv, In review therapeutics, Inc. and Recruitment Partners. AB is a paid consultant for Regeneron Pharmaceuticals and Cognition Therapeutics, Inc and owns equity in Mars Holding Limited. The remaining authors declare that the research was conducted in the absence of any commercial or financial relationships that could be construed as a potential conflict of interest.
