# Supplementary material for: Brain Amyloid Burden and Resting-State Functional Connectivity in Late Middle-Aged Hispanics
Source: Front Neurol. 2020 Oct 6;11:529930. doi: 10.3389/fneur.2020.529930 (PMC7573129; doi:10.3389/fneur.2020.529930)
Supplement: Supplementary file 1 [file Data_Sheet_1.docx]

**Supplemental Table 1.** Nodes location within each functional network

| **Network Name** | **Node** | **MNI Coordinates** | | |
| --- | --- | --- | --- | --- |
|  |  | **X** | **Y** | **Z** |
| DMN | Angular Gyrus (Left) | -42.50 | -69.00 | 31.50 |
| DMN | Angular Gyrus (Right) | 47.33 | -60.33 | 31.00 |
| DMN | Hippocampus (Left) | -24.33 | -39.33 | 7.67 |
| DMN | Hippocampus (Right) | 27.00 | -37.00 | -13.00 |
| DMN | Medial Temporal Gyrus (Left) | -56.57 | -19.14 | -13.57 |
| DMN | Medial Temporal Gyrus (Right) | 56.00 | -4.40 | -20.80 |
| DMN | Orbital Medial Prefrontal Cortex | 2.57 | 43.57 | -7.43 |
| DMN | Precuneus/Posterior Cingulate Cortex | 2.12 | -52.62 | 26.12 |
| DMN | Superior Medial Prefrontal Cortex | -2.74 | 45.58 | 29.74 |
| DAN | Frontal Eye Field (Left) | -32.00 | -1.00 | 54.00 |
| DAN | Frontal Eye Field (Right) | 29.00 | -5.00 | 54.00 |
| DAN | Intraparietal Sulcus (Left) | -25.67 | -58.67 | 49.33 |
| DAN | Intraparietal Sulcus (Right) | 19.00 | -61.67 | 56.33 |
| DAN | Middle Temporal Gyrus (Left) | -47.00 | -61.50 | -2.00 |
| DAN | Middle Temporal Gyrus (Right) | 46.00 | -59.00 | 4.00 |
| FPC | Dorsal Lateral Prefrontal Cortex (Left) | -39.40 | 11.00 | 39.20 |
| FPC | Dorsal Lateral Prefrontal Cortex (Right) | 40.00 | 19.00 | 41.00 |
| FPC | Frontal Cortex (Left) | -39.33 | 46.00 | 7.67 |
| FPC | Frontal Cortex (Right) | 37.20 | 40.20 | 3.60 |
| FPC | Intraparietal Lobule (Left) | -47.50 | -52.00 | 44.00 |
| FPC | Intraparietal Lobule (Right) | 50.33 | -49.33 | 26.00 |
| FPC | Intraparietal Sulcus (Left) | -28.00 | -58.00 | 48.00 |
| FPC | Intraparietal Sulcus (Right) | 35.00 | -59.00 | 42.00 |
| FPC | Paracingulate Gyrus | -3.00 | 26.00 | 44.00 |
| SAL | Anterior Insula (Left) | -35.00 | 20.00 | 0.00 |
| SAL | Anterior Insula (Right) | 35.00 | 19.00 | -2.50 |
| SAL | Anterior Cingulate Gyrus | 0.60 | 23.20 | 32.00 |
| SAL | Frontal Pole (Left) | -33.50 | 51.50 | 19.00 |
| SAL | Frontal Pole (Right) | 29.33 | 46.33 | 23.33 |
| SAL | Inferior Frontal Gyrus (Right) | 42.50 | 27.00 | 4.00 |
| SAL | Precentral Gyrus (Right) | 42.00 | 0.00 | 47.00 |
| SAL | Precuneus | 11.00 | -39.00 | 50.00 |
| SAL | Supra-marginal Gyrus (Right) | 55.00 | -45.00 | 37.00 |


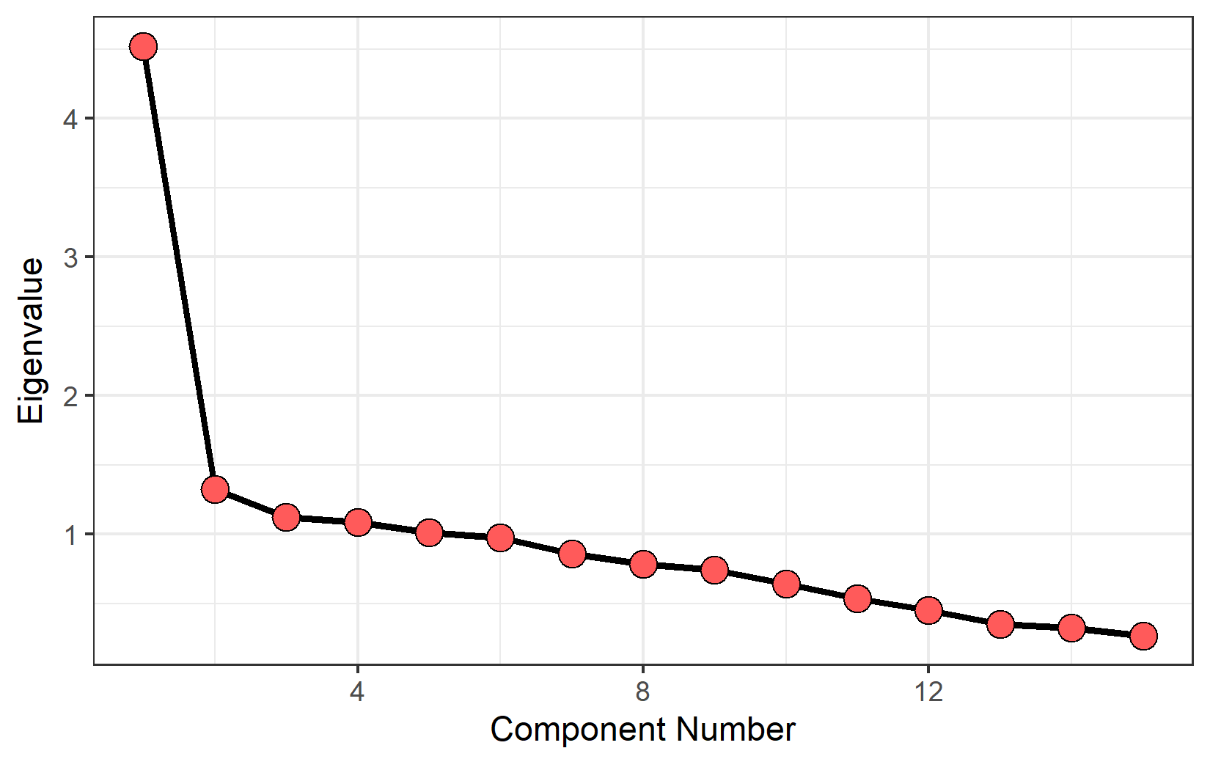


**Suplemental Figure 1. Scree plot for principle component analysis (PCA) of our neuropsychological battery**. Each principle component is plotted sequentially with its corresponding eigenvalue, a value proportional to the amount of variance in the neuropsychological results captured by each individual principle component.

**
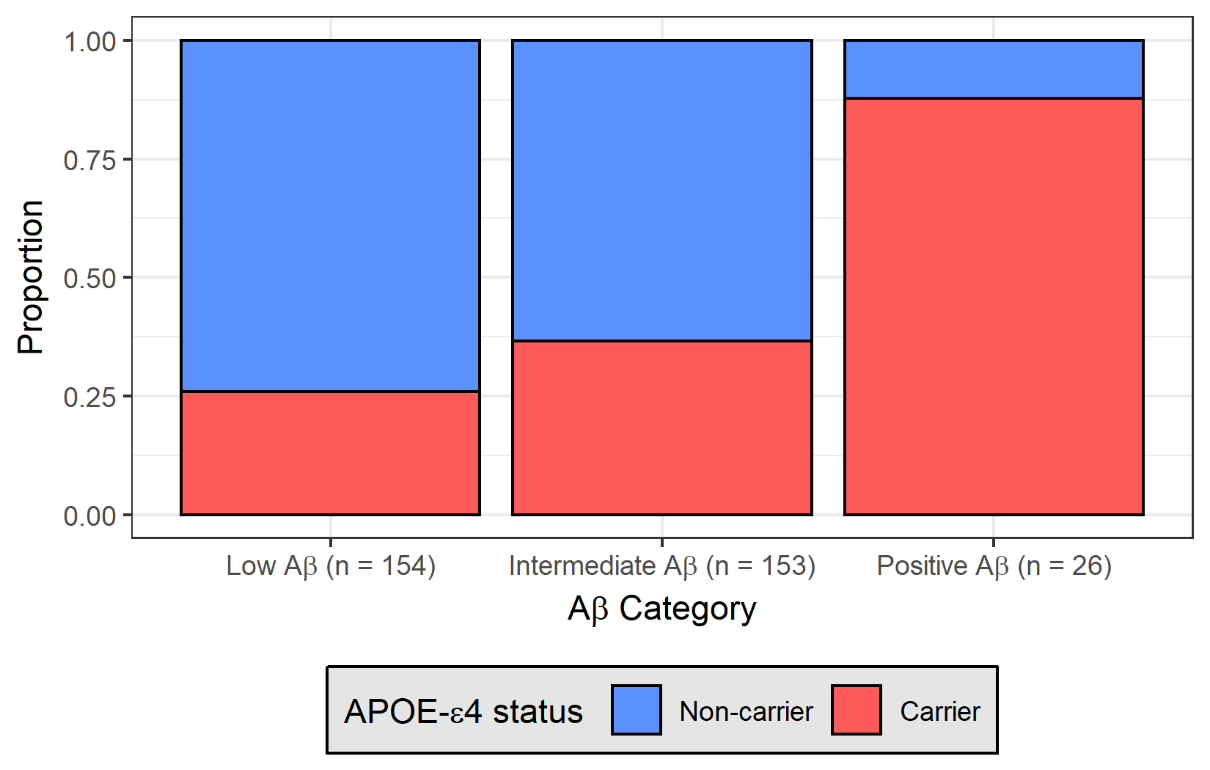
**

**Supplemental Figure 2**. Stacked bar chart representing the percent of APOE-ε4 carriers vs APOE-ε4 non-carriers among amyloid beta (Aβ) categories. Compared to participants without the APOE-ε4 allele, APOE-ε4 carriers had higher odds of being in the intermediate or positive category as compared to the low Aβ category.
